# Genomic Characterization of *Salmonella* Isangi: A Global Perspective of a Rare Serovar

**DOI:** 10.3390/antibiotics12081309

**Published:** 2023-08-11

**Authors:** Anamaria Mota Pereira dos Santos, Pedro Panzenhagen, Rafaela G. Ferrari, Ana Carolina S. de Jesus, Ana Beatriz Portes, Alan Clavelland Ochioni, Dalia dos Prazeres Rodrigues, Carlos Adam Conte-Junior

**Affiliations:** 1 Center for Food Analysis (NAL), Technological Development Support Laboratory (LADETEC), Federal University of Rio de Janeiro (UFRJ), Rio de Janeiro 21941-598, RJ, Brazil; anamariasantos@id.uff.br (A.M.P.d.S.); rafaelaferrari@yahoo.com.br (R.G.F.); carolana08@outlook.com (A.C.S.d.J.); aportes@id.uff.br (A.B.P.); alanreaver@yahoo.com.br (A.C.O.); conte@iq.ufrj.br (C.A.C.-J.); 2 Laboratory of Advanced Analysis in Biochemistry and Molecular Biology (LAABBM), Department of Biochemistry, Federal University of Rio de Janeiro (UFRJ), Rio de Janeiro 21941-909, RJ, Brazil; 3 Graduate Program in Veterinary Hygiene (PGHIGVET), Faculty of Veterinary Medicine, Fluminense Federal University (UFF), Niterói 24230-340, RJ, Brazil; 4 Graduate Program in Food Science (PPGCAL), Institute of Chemistry (IQ), Federal University of Rio de Janeiro (UFRJ), Rio de Janeiro 21941-909, RJ, Brazil; 5 Laboratory of Enterobacteria (LABENT), Fundação Oswaldo Cruz—FIOCRUZ, Rio de Janeiro 21040-900, RJ, Brazil; dalia@ioc.fiocruz.br; 6 Graduate Program in Sanitary Surveillance (PPGVS), National Institute of Health Quality Control (INCQS), Oswaldo Cruz Foundation (FIOCRUZ), Rio de Janeiro 21040-900, RJ, Brazil

**Keywords:** whole-genome sequencing, nontyphoidal *Salmonella*, multidrug resistance, ESBL, PMQR, quinolone, beta-lactamase

## Abstract

*Salmonella* Isangi is an infrequent serovar that has recently been reported in several countries due to nosocomial infections. A considerable number of reports indicate *Salmonella* Isangi multidrug resistance, especially to cephalosporins, which could potentially pose a risk to public health worldwide. Genomic analysis is an excellent tool for monitoring the emergence of microorganisms and related factors. In this context, the aim of this study was to carry out a genomic analysis of *Salmonella* Isangi isolated from poultry in Brazil, and to compare it with the available genomes from the Pathogen Detection database and Sequence Read Archive. A total of 142 genomes isolated from 11 different countries were investigated. A broad distribution of extended-spectrum beta-lactamase (ESBL) genes was identified in the *Salmonella* Isangi genomes examined (*bla*_CTX-M-15_, *bla*_CTX-M-2_, *bla*_DHA-1_, *bla*_NDM-*1*_, *bla*_OXA-10_, *bla*_OXA-1_, *bla*_OXA-48_, *bla*_SCO-1_, *bla*_SHV-*5*_, *bla*_TEM-131_, *bla*_TEM-1B_), primarily in South Africa. Resistome analysis revealed predicted resistance to aminoglycoside, sulfonamide, macrolide, tetracycline, trimethoprim, phenicol, chloramphenicol, and quaternary ammonium. Additionally, PMQR (plasmid-mediated quinolone resistance) genes *qnr19*, *qnrB1*, and *qnrS1* were identified, along with point mutations in the genes *gyrA*^D87N^, *gyrA*^S83F^, and *gyrB*^S464F^, which confer resistance to ciprofloxacin and nalidixic acid. With regard to plasmids, we identified 17 different incompatibility groups, including IncC, Col(pHAD28), IncHI2, IncHI2A, IncM2, ColpVC, Col(Ye4449), Col156, IncR, IncI1(Alpha), IncFIB (pTU3), Col(B5512), IncQ1, IncL, IncN, IncFIB(pHCM2), and IncFIB (pN55391). Phylogenetic analysis revealed five clusters grouped by sequence type and antimicrobial gene distribution. The study highlights the need for monitoring rare serovars that may become emergent due to multidrug resistance.

## 1. Introduction

Nontyphoidal *Salmonella* (NTS) remains one of the most critical enterobacteria, causing foodborne gastroenteritis worldwide (WHO, 2022). *Salmonella* Typhimurium and *Salmonella* Enteritidis continue to be the most reported NTS serovars due to their high endemicity in numerous countries [1,2]. *Salmonella enterica* encompasses approximately 2610 different serovars, many of which are rare and neglected [2]. However, the emergence of rare serovars in human and animal populations should not be ignored. Recently, rare serovars have emerged in African [3], American [4], and Asian [5] countries, with many exhibiting multidrug resistance to antimicrobials. Horizontal gene transfer (HGT) plays a crucial role in these emergences, primarily with plasmids, which can be easily transmitted among serovars [1]. Therefore, monitoring and surveillance of all *Salmonella* serovars become essential to predict and understand the dissemination trends of rare serovars and to develop prevention measures against emerging pathogens. 

*Salmonella* Isangi is a less-known serovar with few attributed studies. Extended-spectrum beta-lactamase (ESBL)-producing *Salmonella* Isangi has become common in South Africa and has also been reported in several other countries, mainly in association with nosocomial infections [3,4,6,7,8]. Notably, Brazil appears to be the only country reporting *Salmonella* Isangi in animal production, particularly, poultry [9], which may represent a new trend in this serovar adaptation in the last few years. The emergence of this serovar in animal production represents a risk for the One Health approach. 

Large-scale genomic analysis has become a fundamental epidemiological tool in collecting and exploring free available complete genomes in several public databases [10,11,12]. Critical information may go unnoticed when many samples are sequenced for surveillance purposes, and many genomes remain unexplored. Thus, large-scale genomic analysis can be a solution to better understand the distribution of genes and topographical characteristics such as location, species, and primary sources of isolation of pathogens not usually studied [12,13,14,15].

As *Salmonella* Isangi seems to be emerging and adapting within Brazilian poultry production, we sought to perform a genomic characterization of a recently sequenced isolate from poultry production in Brazil. In parallel, we genetically compared it with other already available *Salmonella* Isangi genomes in the NCBI database worldwide to bring new understanding about the genetic epidemiology of this rare serovar.

## 2. Material and Methods

### 2.1. Bacterial Isolate and Whole-Genome Sequencing

The *Salmonella* Isangi isolate was obtained from the reference laboratory of enterobacteria of the Oswaldo Cruz Institute (LABENT-IOC) *Salmonella* collection. DNA extraction was performed using a commercial kit (Dneasy Blood & Tissue, QIAGEN, Hilden, Germany) according to the manufacturer’s instructions. The genomic DNA of the isolate was sequenced using the Illumina DNA prep library preparation kit at the Miseq platform (Illumina, San Diego, CA, USA) with 300 bp paired-end sequencing. 

### 2.2. Acquisition of Complementary Genomic Data 

The filtering tools on the Sequence Read Archive (SRA) web browser of the National Center for Biotechnology Information (NCBI) database (https://www.ncbi.nlm.nih.gov/pathogens/ (accessed on 2 August 2022)) were used to select and download raw read sequences of *Salmonella* Isangi available as of 2 August 2022. The genomes were selected according to their quality (i.e., N50 and contig number) and metadata availability (i.e., isolation source, collection date, location). 

### 2.3. Genome Assembly and Quality Filtering

Trimmomatic 0.39 [16] was used to trim raw sequence reads and remove poor-quality bases. FastQC v0.11.9 was employed to assess the quality of trimmed reads and SPAdes 3.15.4 [17] for de novo assembly. The quality of draft genomes was evaluated using QUAST v5.2.0.0 [18] and the assembled contigs were ordered using Mauve 2.4.0 [19]. Samtools 1.15.1 was used to estimate the assembled average coverage. All sequences were annotated using Prokka version 1.14.6 [20]. 

### 2.4. In Silico Genome Characterization

SISTR (*Salmonella* in silico Typing Resource) 1.1.1 [21] software was used to perform in silico serotyping on all the *Salmonella* Isangi genomes. Multi-locus sequence typing (MLST) software 2.19.0 [22] was employed for in silico genotyping. To identify antimicrobial resistance genes (AMR), plasmid replicons, and virulence factors, ABRicate software 1.0.1 (https://github.com/tseemann/abricate (accessed on 24 January 2023)) was used alongside the Resfinder [23], Plasmidfinder [24], and Virulence Factor (VFDB) (http://www.mgc.ac.cn/VFs/ (accessed on 24 January 2023)) databases, respectively. Pointfinder software 0.8.0 [23] was used to identify AMR point mutations conferring potential antimicrobial resistance within the genomes. A customized pESI database [13] was used to identify plasmids for emergent *Salmonella* Infantis on the *Salmonella* Isangi genomes. Minimum nucleotide identity and coverage thresholds of 90% and 60%, respectively, were employed for all analyses. 

### 2.5. Phylogenetic Analysis

The detection and prediction of the effects of whole-genome variants were carried out using the Rapid haploid variant calling and core genome alignment—Snippy v4.6.0 (https://github.com/tseemann/snippy (accessed on 24 January 2023)). As there is no complete sequence of *Salmonella* Isangi available as a reference in the public genome repertoires, we used a complete genome from *Salmonella* Infantis (Accession number GCA_029592185.1), which was also our outgroup. Gubbins v2.4.1 was used to identify and exclude recombinant regions within the core genome alignment to enhance the accuracy of phylogenetic reconstructions. Phastaf v0.1.0 (https://github.com/tseemann/phastaf (accessed on 24 January 2023)) and Barrnap v0.9 (https://github.com/tseemann/barrnap (accessed on 24 January 2023)) were used to identify phage regions and ribosomal RNA for masking purposes in the reference genome, respectively. The final high-quality core-genome SNPs were extracted from the alignment file using the software SNP-sites v2.5.1 [25]. Iqtree v2.0.3 was employed to generate a maximum likelihood phylogeny from this core genome alignment, using a GTR + F + I + G4 substitution model, with 1000 bootstraps replication to support the tree nodes.

## 3. Results

### 3.1. Data Collection and MLST Distribution

A total of 141 raw sequences of *Salmonella* Isangi were downloaded from the SRA web browser. By utilizing the SISTR software, we confirmed that all genomes were Isangi serovar. The genomes were obtained from strains isolated from 11 different countries: South Africa (n = 65); Uganda (n = 1); United Kingdom (n = 35); United States of America (n = 16); Brazil (n = 15); Germany (n = 1), India (n = 2), Netherlands (n = 1), Ireland (n = 2), Indonesia (n = 1), and Taiwan (n = 1) (Supplementary Table S1). Our analysis comprised 142 genomes, including the newly sequenced *Salmonella* Isangi genome (Sal08932019 Accession: JARETA000000000) from Brazil. Regarding the sources, 109 genomes came from human isolates, three were from animals, three from food, seven from feed, six from the environment, and 14 were not identified. We identified that genomes belonged to nine different sequence types (ST): ST216 (n = 62); ST335 (n = 59); ST1994 (n = 8); ST2261 (n = 1); ST369 (n = 1); ST3101 (n = 1); ST3390 (n = 1); ST5028 (n = 1); and ST7036 (n = 2) (Supplementary Table S1).

### 3.2. Resistome

**Figure 1:**
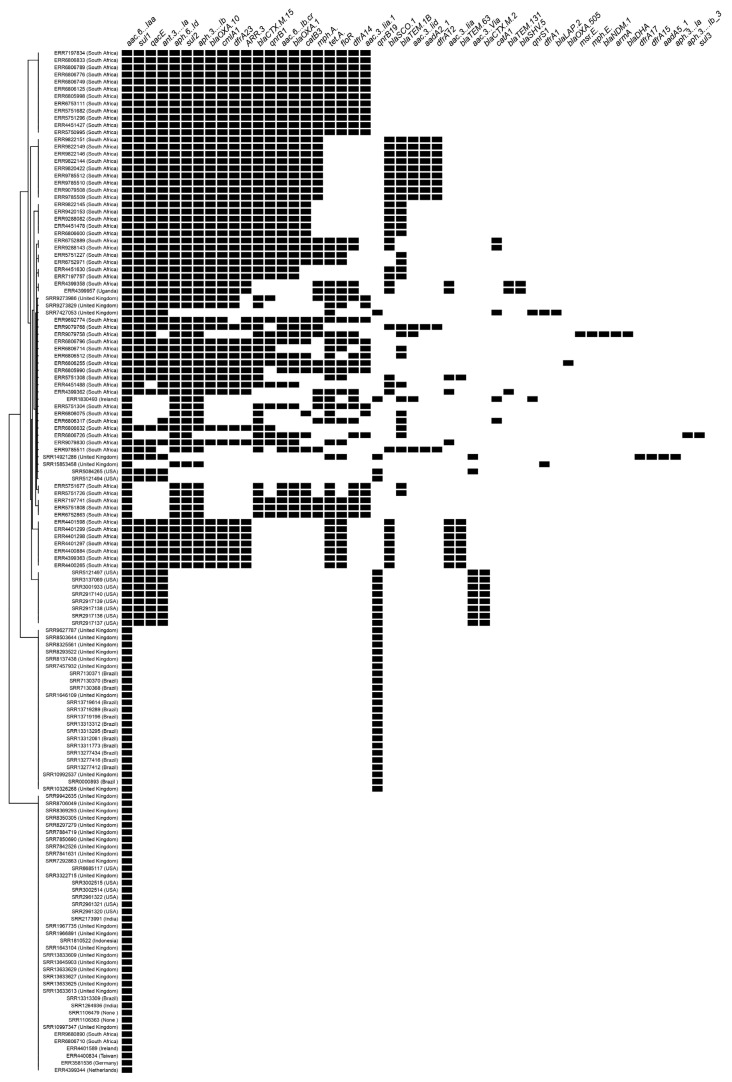
Predicted genetic traits of the *Salmonella* Isangi (n = 142). AMR genes presence is represented by the black spots.

The 142 genomes presented a resistome that included the following repertoire of antimicrobial genes: aminoglycoside [*aac(3)-Iia_1, aac(3)-Iid, aac(3)-Via, aac(6’)-Iaa, aac(6’)-Ib-cr, aadA1, aadA2,aadA5, ant(3’’)-Ia, aph(3’’)-Ib, aph(3’’)-Ib, aph(3’)-Ia, aph(6)-Id*], sulfonamide [*sul*1, *sul*2, *sul*3], macrolide [*mph*(A), *mph*(E)], tetracycline [*tet*(A)], trimethoprim [*dfr*A12, *dfr*A14, *dfr*A15, *dfr*A17, *dfr*A1, *dfr*A23], phenicol [*flo*R], beta-lactams [*bla*_CTX-M-15_, *bla*_CTX-M-2_, *bla*_DHA-1_, *bla*_NDM-*1*_, *bla*_OXA-10_, *bla*_OXA-1_, *bla*_OXA-48_, *bla*_SCO-1_, *bla*_SHV-*5*_, *bla*_TEM-131_, *bla*_TEM-1B_], chloramphenicol [*cat*A1, *cat*B3], and quaternary ammonium [*qac*E] (Supplementary Table S1). We identified a wide distribution of extended-spectrum beta-lactamase (ESBL) genes among the genomes from South Africa and the United Kingdom (Figure 1). ESBL genes were also identified in *Salmonella* Isangi isolated from Ireland (*bla*_TEM-1B_) and the United States (*bla*_CTX-M-2_). Furthermore, we detected 83 genomes exhibiting plasmid-mediated quinolone resistance (PMQR) genes (Supplementary Table S1). The PMQR *qnrB1* (n = 47) was the most common quinolone resistance gene observed, followed by *qnrB19* (n = 36) and *qnrS1* (n = 2). All 47 genomes carrying the *qnrB1* gene co-produced at least one ESBL gene (*bla*_CTX-M-15_, *bla*_DHA-1_, *bla*_NDM-1_, *bla*_OXA-10_, *bla*_OXA-1_, *bla*_OXA-48_, *bla*_SCO-1_, *bla*_SHV-5_, *bla*_TEM-131_, *bla*_TEM-1B_), with many of them co-producing more than one. Ten of the genomes carrying the *qnrB19* gene co-produced ESBL gene (*bla*_CTX-M-*2*_) and were isolated from the United States, whereas the others were positive only for *qnrB19*. All genomes carrying *qnrS1* were positive for *qnrB19* and co-produced ESBL genes (*bla*_LAP-2_, *bla*_TEM-1B_). Our sequenced genome (Sal08932019 Accession: JARETA000000000) presented only the plasmid-mediated quinolone resistance gene *qnrB19*, a characteristic shared with the other *Salmonella* Isangi isolated from Brazil [9]. The PMQR *qnrB19* was identified in genomes isolated from Brazil (n = 14), the United Kingdom (n = 11), the United States (n = 10), and Ireland (n = 1). The *qnrB1* was primarily identified in South Africa (n = 46) and the United Kingdom (n = 1). The *qnrS1* was identified in genomes isolated from Ireland (n = 1) and the United Kingdom (n = 1). Regarding the determination of quinolone resistance, 10 isolates presented a single mutation in the genes *gyrA*^D87N^, *gyrA*^S83F^, and *gyrB*^S464F^, which conferred resistance to ciprofloxacin and nalidixic acid. These genomes were all isolated from South Africa (Table 1). Seven genomes isolated from the United Kingdom (n = 6) and India (n = 1) presented a single mutation in the gene *parC*^T57S^; however, there was no predicted phenotype.

### 3.3. Virulome

The analyzed genomes presented the core virulence genes responsible for *Salmonella*’s pathogenicity (Supplementary Table S1). Moreover, three genomes from South Africa (n = 2) and the United States (n = 1) presented the yersiniabactin siderophore (*ybtAPQSTUX*, *fyuA*, *irp1*, and *irp2*), which increases iron uptake. We also evaluated the presence of pESI using a customized database with the pESI backbone genes. A single genome from India presented 99 out of 113 genes from the pESI core genome. This genome did not display any antimicrobial resistance genes or other essential genes that make up the pESI megaplasmid. Beforehand, we ruled out the hypothesis that this genome had a specific pESI structure.

The most frequent plasmid incompatibility group identified among the analyzed *Salmonella* Isangi was IncC [n = 66], followed by Col(pHAD28) [n = 39], IncHI2 [n = 37], IncHI2A [n = 28], IncM2 [n = 12], ColpVC [n = 6], Col(Ye4449) [n = 5], Col156 [n = 5], IncR [n = 5], IncI1(Alpha) [n = 4], IncFIB (pTU3) [n = 3], Col(B5512) [n = 2], IncQ1 [n = 2], IncL [n = 2], IncN [n = 2], IncFIB(pHCM2) [n = 1], and IncFIB (pN55391) [n = 1]. 

All genomes carrying the PMQR *qnrB19* mapped to the Col(pHAD28) incompatibility group, with different distributions of other incompatibility groups [Col(Ye4449), ColpVC, and IncL] (Supplementary Table S2). Among the genomes that co-produced the ESBL gene *bla*_CTX-M-2_, we identified the incompatibility groups IncHI2, IncHI2A, and IncQ1. All genomes carrying the PMQR *qnrB1* displayed the IncC incompatibility group, with different distributions of other incompatibility groups [IncCM2, IncHI2, IncHI2A, IncI1(Alpha), IncL, and IncFIB (pHCM2)]. The genomes carrying the *qnrS1* displayed the same distribution of incompatibility groups as the genomes carrying the *qnrB19* gene (Supplementary Table S2).

### 3.4. Phylogenetics and Characterization of Salmonella Isangi Lineages 

**Figure 2:**
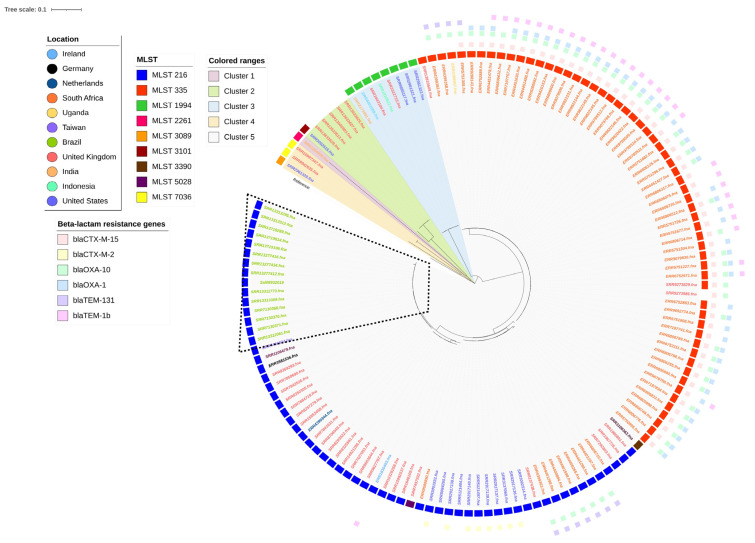
Maximum-likelihood SNP-based phylogeny of 142 *Salmonella* Isangi genomes using, as reference, *Salmonella* Infantis (accession number GCA_029592185.1). The circular representation of the phylogeny was obtained using iTOL (http://itol.embl.de/ (accessed on 8 February 2023)), ignoring branch lengths. Colors of the isolates’ ID indicate different countries of origin. Colored squares indicate the genome sequence type (MLST) and the beta-lactam resistance gene presence. Isolates from Brazil are detached.

We conducted a whole-genome variant detection on the set of 142 *Salmonella* Isangi genomes. The phylogenetic tree that was generated showed five well-defined clusters, mostly grouped according to country, ESBL gene distribution, and MLST (Figure 2). Cluster 1 included a single genome isolated from India (ST2261) with no antimicrobial resistance gene. Cluster 2 differed from the other clusters by 13,601 SNPs and consisted of six genomes: one from the United States and five from the United Kingdom. Cluster 3 differed from the other clusters by 22,877 SNPs and was composed of three genomes: one from the United States (ST3059) and two from the United Kingdom (ST7036). Cluster 4 comprised eight genomes within the ST1994 and differed from the other clusters by 28,422 SNPs. Three came from the United States, two from the United Kingdom, and one from each of India, Ireland, and Indonesia. Cluster 5 was defined on each from ST216 and ST335, the most frequent MLST among the studied genomes, and differed from the other clusters by 22,877 SNPs. They were grouped by country, and the ESBL distribution also contributed to the genome clustering. All the genomes from South Africa presented at least one ESBL gene, with *bla*_CTX-M-15_ being the most frequent. A single ST335 genome from the United Kingdom was grouped with South African genomes and did not present the ESBL gene. The ST216 genomes from the United States were grouped beside and presented *bla*_CTX-M-2_. The ST216 genomes from South Africa showed the ESBL genes *bla*_OXA-10_ and *bla*_TEM-1B_. All the genomes from Brazil belonged to the ST216 and possessed no ESBL genes. However, Brazilian genomes, including ours, presented the *qnrB19* quinolone resistance gene. 

## 4. Discussion

Our literature review identified very few studies on *Salmonella* Isangi in Brazil and worldwide [3,4,9,26,27]. Recently, this serovar has been associated with foodborne disease outbreaks in China and nosocomial infections in the United States and South Africa [3,4,5,9,26,27], particularly those caused by *Salmonella* Isangi with ESBL genes. The emergence of rare serovars with antimicrobial resistance or multidrug resistance poses a risk to public health, as they tend to be neglected due to inadequate monitoring and prevention efforts. In this study, we performed genomic characterization of a *Salmonella* Isangi strain isolated from poultry in Brazil in association with other *Salmonella* Isangi genomes available in the NCBI database, to provide new insights from a global perspective.

The majority of the genomes analyzed in this study were isolated from South Africa (Figure 2). According to the Enteric Diseases Reference Unit of the National Institute for Communicable Diseases in South Africa, there has been an increase in *Salmonella* Isangi infections in the country since 2000 [6], which explains this high prevalence. We identified genomes isolated from 2001 to 2020, proving that the widespread dissemination of *Salmonella* Isangi is still occurring in the country. It is worth noting, however, that our results are limited to genomes that have been sequenced and deposited in the NCBI database. Nevertheless, these results may reflect a broader reality of emergence of *Salmonella* Isangi in South Africa. South Africa has the most significant number of HIV-positive patients worldwide [28], who tend to develop severe non-typhoidal *Salmonella* infection due to their immunodeficient status [29]. The high demand for last-choice antimicrobials, including extended-spectrum cephalosporins [6] to treat these severe infections, may contribute to the emergence of ESBL-positive *Salmonella* Isangi in the South African population. 

Of the South African genomes analyzed, 54 (83.1%) displayed the *bla*_CTX-M-15_ gene. *Salmonella* Isangi containing this resistance gene [4] has been previously reported in a hospital outbreak in the United States. Initially, *bla*_CTX-M-15_ was associated exclusively with *E. coli* and *Klebsiella* spp. in the human population. However, recent epidemiological studies have reported the CTX-M-15 variant as one of the most widely dominant CTX-M β-lactamases [30,31]. The pandemic dissemination of CTX-M-15 among *Enterobacteriaceae* originated from a highly virulent *E. coli* O25:H4-ST131 [32,33], and their widespread occurrence is linked to the insertion element IS*Ecp*I responsible for the mobilization of *bla* genes [34]. We evaluated the presence of IS*Ecp*I in the South African genomes with *bla*_CTX-M-15._ All presented this mobile element, confirming that the horizontal gene transfer ability among the *Salmonella* Isangi is already established in the South African population. The *bla*_CTX-M-15_ gene is currently associated with the *bla*_TEM-1_ and *bla*_OXA-1_ resistance genes [32], and was also detected in most South African genomes (Figure 1). Although this association is often related to plasmids belonging to the IncF group [32], we observed a strong association of these genes with the IncC group in our study. Previous research conducted on *E. coli* demonstrated that the IncF CTX-M-15 plasmid is better adapted to this species due to its lower fitness cost compared to the IncC CTX-M-15 plasmid [31]. However, selective pressure may have been the crucial factor for the rapid adaptation of IncC plasmid among bacteria [31], and may have occurred in *Salmonella* Isangi genomes from South Africa.

Genomes belonging to ST335 presented *qnrB1* that confers low-level quinolone resistance [35]. The Qnr family of proteins is known to protect DNA gyrase and topoisomerase from quinolone antimicrobial activity [35] and to facilitate the selection of high-level quinolone-resistant mutants [35]. The presence of PMQR genes such as *qnrB1* has raised concerns among health authorities in recent years precisely because of their potential to increase resistance among bacteria. Because they are plasmodial genes, their spread is facilitated by horizontal gene transfer [36]. Therefore, active surveillance of these genes is essential to prevent the spread and emergence of serovars, such as *Salmonella* Isangi, which are not commonly associated with severe infection episodes. The high incidence of ST335 in South Africa, with multiple ESBL genes alongside the PMQR, may justify its endemic status across Africa. We also identified *Salmonella* Isangi ST335 in Uganda and the United Kingdom, which were positive for ESBL genes and more closely related to the South African genomes (Figure 1). These results reiterate the possibility of rare serovars becoming epidemic and that, therefore, they should not be neglected. 

ST216 was the most widespread sequence type in the United States, the United Kingdom, South Africa, and Brazil. This sequence type has recently been found in Africa and Asia [5,37]. Among the 62 ST216 genomes, 56.5% (35) presented the PMQR gene *qnrB19*. The presence of *qnrB19* dated from 2012 is relatively recent compared to genomes isolated before (2001–2008) (Supplementary Table S1). These results are compatible with the first description of *qnrB19* in *E. coli* isolated from pigs in China in 2008 [38]. Quinolone is a widespread group of antimicrobials used to treat human and livestock animal infection [39]. Therefore, the cross-transfer of resistance genes from animals to humans could be facilitated. Several studies have linked the presence of *qnrB19* to the increase of quinolone resistance in some South American countries, the United States, and Europe [40,41,42]. *Salmonella* Isangi presenting this PMQR gene in Brazil has been already studied [9], although the presence of *qnrB19* in other *Salmonella* serovars has also been reported [43,44,45]. Among the ST216, only the Brazilian genomes were isolated from poultry or poultry environment. Our sequenced genome (Sal08932019 Accession: JARETA000000000) displayed the same genotype as the other genomes previously sequenced from Brazil, indicating that *Salmonella* Isangi in Brazil is likely emerging recently from abroad. The presence of *qnrB19* in *Salmonella* Isangi is a concern for health authorities and threatens One Health maintenance. The gradual reduction of endemic *Salmonella*, such as *S.* Typhimurium and *S.* Enteritidis, in animal production as a result of vaccination may have created opportunities for the emergence of rare *Salmonella* serovars, such as *Salmonella* Isangi, in the food production chain, as observed in Brazil. Hence, monitoring resistance genes and plasmids is strategic to prevent and control their emergence in rare serovars.

Previous studies have identified an increased phenotypic and genotypic resistance to quinolones in *Salmonella* Isangi isolated from poultry [9,46]. Most of the analysis relates the quinolone resistance to two determinants: multiples point mutations on the quinolone resistance-determining region (QRDR) of DNA gyrase (*gyrA*) and the topoisomerase C (*parC*) (predicted to provide ciprofloxacin and nalidixic acid resistance) [47], and PMQR. In our study, only 10 of the analyzed genomes presented QNDR point mutations (*gyrA*^D87N^, *gyrA*^S83F^, and *gyrB*^S464F^), all of which were isolated from South Africa in 2001. In contrast, seven genomes from the United Kingdom and India presented point mutation (*parC*^T57S^) and were isolated in recent years, indicating that this specific point mutation is more widespread within recent *Salmonella* Isangi. No relation between QRDR and PMQR was observed.

Eight *Salmonella* Isangi genomes from the United States belonging to ST216 displayed *bla*_CTX-M-2_. In the United States, *bla*CTX-M-producing *Salmonella* Isangi were associated with hospital outbreaks [4]. However, to our knowledge, this is the first report of *bla*_CTX-M-2_ in *Salmonella* Isangi isolated from the United States. The genomes described in this study were available in 2015, but, so far, we have not identified studies concerning the CTX-M-2 in *Salmonella* spp. in the United States. However, several recent studies in Brazil, Colombia, and South Africa have identified CTX-M-2 in various *Salmonella* serovars associated with the poultry industry [48,49,50,51]. As reviewed by Bevan et al. (2017) [30], CTX-M-2 enzyme was the primary genotype in South America and was the only one identified before the 1990s [52]. This incidence remains high in the Americas, as evidenced by studies conducted mainly in *E. coli* [53,54,55]. CTX-M-2 seems to be highly associated with the poultry industry, which may pose a field condition for their rapid spread among other countries through horizontal gene transfer. Our results demonstrate the importance of constantly monitoring genomes deposited in databases such as the NCBI. 

*Salmonella* Isangi genomes were grouped based on the country and ST profile (Figure 2). The phylogenetic analysis revealed five different clades. The main difference among these clades is the distribution of beta-lactam resistance genes, which we observed to be more widespread in the South African population. The most phylogenetically distant genome was from India and was clustered alone in Cluster 1. It displayed a differentiated distribution of virulence genes, with 99 out of 113 genes from the pESI megaplasmid core genome. According to Hall et al. (2022) [56] forming megaplasmids such as pESI requires the imposition of selective pressure from different niches until the megaplasmid is developed, allowing the bacteria to survive in this challenging environment. Although our analysis demonstrated no pESI in the Indian genome, these results suggest only the beginning of the acquisition of genes, due to the different selective pressures imposed in the Indian isolate. Further genomic studies are required to corroborate this hypothesis. 

Regarding virulence genes, three genomes from South Africa (2020) and the United States (2010) presented the complete yersiniabactin siderophore (*ybtAPQSTUX*, *fyuA*, *irp1*, *and irp2*) that increases iron uptake. These genes are part of the *Yersinia* High Pathogenicity Island (HPI) [57], which is commonly found in *Yersinia*, *Salmonella* spp., and *E. coli* [57,58]. The presence of HPI in enterobacteria has been shown to increase environmental persistence and fitness [59]. This study suggests that the acquisition of HPI may be a determinant for *Salmonella* Isangi dissemination and persistence across these countries and the established population.

## 5. Conclusions

In summary, our study highlights the endemic success of *Salmonella* Isangi in South Africa and how it could be related to the acquisition of multiple ESBL genes associated with PMQR genes, mainly *qnrB1*. These results reaffirm the need to constantly monitor *Salmonella* Isangi and other rare serovar dissemination across continents, as MDR *Salmonella* spp. pose a significant risk to public health. Moreover, the presence of antimicrobial-resistant *Salmonella* Isangi in the Brazilian poultry industry could significantly impact One Health, given the international importance of Brazil in the production and export of poultry products and their potential risk of transmission to humans. Lastly, our results highlight the importance of large-scale genomic analysis as an epidemiological and monitoring tool for the surveillance of *Salmonella* and other microorganisms. 

## Figures and Tables

**Table 1:** Features of *Salmonella* Isangi (n = 15) displaying point mutation in *gyrA* and *parC* genes, conferring resistance to ciprofloxacin and nalidixic acid.

Isolate ID	Location	Year of Isolation	Resistome	Gene	Mutation	Predicted Phenotype
ERR4399363	South Africa	2001	*aac(3)-IIa*, *ant(3″)-Ia*, *aph(3″)-Ib*, *aph(6)-Id*, *ARR-3*, *blaOXA-10*, *blaSCO-1*, *blaTEM-63*, *cmlA1*, *dfrA23*, *floR*, *gyrA (D87N)*, *qacE*, *sul1*, *sul2*, *tet(A)*	*gyrA* (D87N)	GAC→AAC (D→N)	ciprofloxacin I/R, nalidixic acid
ERR4400265	South Africa	2001	*aac(3)-IIa*, *ant(3″)-Ia*, *aph(3″)-Ib*, *aph(6)-Id*, *ARR-3*, *blaOXA-10*, *blaSCO-1*, *blaTEM-63*, *cmlA1*, *dfrA23*, *floR*, *gyrA (D87N)*, *qacE*, *sul1*, *sul2*, *tet(A)*	*gyrA* (D87N)	GAC→AAC (D→N)	ciprofloxacin I/R, nalidixic acid
ERR4400834	Tawain	2007		*gyrA* (S83F)	TCC→TTC (S→F)	ciprofloxacin I/R, nalidixic acid
ERR4400884	South Africa	2001	*aac(3)-IIa*, *ant(3″)-Ia*, *aph(3″)-Ib*, *aph(6)-Id*, *ARR-3*, *blaOXA-10*, *blaSCO-1*, *blaTEM-63*, *cmlA1*, *dfrA23*, *floR*, *gyrA (D87N)*, *qacE*, *sul1*, *sul2*, *tet(A)*	*gyrA* (D87N)	GAC→AAC (D→N)	ciprofloxacin I/R, nalidixic acid
ERR4401297	South Africa	2001	*aac(3)-IIa*, *ant(3″)-Ia*, *aph(3″)-Ib*, *aph(6)-Id*, *ARR-3*, *blaOXA-10*, *blaSCO-1*, *blaTEM-63*, *cmlA1*, *dfrA23*, *floR*, *gyrA (D87N)*, *qacE*, *sul1*, *sul2*, *tet(A)*	*gyrA* (D87N)	GAC→AAC (D→N)	ciprofloxacin I/R, nalidixic acid
ERR4401298	South Africa	2001	*aac(3)-IIa*, *ant(3″)-Ia*, *aph(3″)-Ib*, *aph(6)-Id*, *ARR-3*, *blaOXA-10*, *blaSCO-1*, *blaTEM-63*, *cmlA1*, *dfrA23*, *floR*, *gyrA (D87N)*, *qacE*, *sul1*, *sul2*, *tet(A)*	*gyrA* (D87N)	GAC→AAC (D→N)	ciprofloxacin I/R, nalidixic acid
ERR4401299	South Africa	2001	*aac(3)-IIa*, *ant(3″)-Ia*, *aph(3″)-Ib*, *aph(6)-Id*, *ARR-3*, *blaOXA-10*, *blaSCO-1*, *blaTEM-63*, *cmlA1*, *dfrA23*, *floR*, *gyrA (D87N)*, *qacE*, *sul1*, *sul2*, *tet(A)*	*gyrA* (D87N)	GAC→AAC (D→N)	ciprofloxacin I/R, nalidixic acid
ERR4401598	South Africa	2001	*aac(3)-IIa*, *ant(3″)-Ia*, *aph(3″)-Ib*, *aph(6)-Id*, *ARR-3*, *blaOXA-10*, *blaSCO-1*, *blaTEM-63*, *cmlA1*, *dfrA23*, *floR*, *gyrA (D87N)*, *qacE*, *sul1*, *sul2*, *tet(A)*	*gyrA* (D87N)	GAC→AAC (D→N)	ciprofloxacin I/R, nalidixic acid
ERR6806075	South Africa	2020	*aac(3)-IIa*, *aph(3″)-Ib*, *aph(6)-Id*, *blaCTX-M-15*, *blaTEM-1B*, *catB3*, *gyrB (S464F)*, *sul2*	*gyrB* (S464F)	TCT→TTC (S→F)	ciprofloxacin I/R
SRR1106479	Unknown			*gyrA* (S83F)	TCC→TTC (S→F)	ciprofloxacin I/R, nalidixic acid
SRR1264936	India	2011	*parC (T57S)*	*parC* (T57S)	ACC→AGC (T→S)	None
SRR13633613	United Kingdom	2021	*parC (T57S)*	*parC* (T57S)	ACC→AGC (T→S)	None
SRR13633625	United Kingdom	2021	*parC (T57S)*	*parC* (T57S)	ACC→AGC (T→S)	None
SRR13633627	United Kingdom	2021	*parC (T57S)*	*parC* (T57S)	ACC→AGC (T→S)	None
SRR13633629	United Kingdom	2021	*parC (T57S)*	*parC* (T57S)	ACC→AGC (T→S)	None
SRR13645903	United Kingdom	2021	*parC (T57S)*	*parC* (T57S)	ACC→AGC (T→S)	None
SRR3002515	United States	2009	*parC (T57S)*	*parC* (T57S)	ACC→AGC (T→S)	None

## Data Availability

The data presented in this study are available on the institutional websites cited in the Section 2 and supplementary material.

## Supplementary Materials

The following supporting information can be downloaded at: https://www.mdpi.com/article/10.3390/antibiotics12081309/s1, Supplementary Table S1: Features of all analyzed *Salmonella* Isangi (n = 142), comprising information about location, isolation source, isolation type, year of isolation, SNPcluster, AMR genotype and predicted phenotype, plasmids, cgMLST, and virulence genes’ presence and absence (number 1 represents presence and 0 represents absence); Supplementary Table S2: Features of *Salmonella* Isangi, displaying PMQR genes and distribution of their incompatibility groups.

## References

[1] Tankson J.D., Fedorka-Cray P.J., Jackson C.R., Headrick M. (2006). Genetic relatedness of a rarely isolated Salmonella: Salmonella enterica serotype Niakhar from NARMS animal isolates. J. Antimicrob. Chemother..

[2] Hendriksen R.S., Vieira A.R., Karlsmose S., Lo Fo Wong D.M.A., Jensen A.B., Wegener H.C., Aarestrup F.M. (2011). Global Monitoring of Salmonella Serovar Distribution from the World Health Organization Global Foodborne Infections Network Country Data Bank: Results of Quality Assured Laboratories from 2001 to 2007. Foodborne Pathog. Dis..

[3] Govinden U., Mocktar C., Moodley P., Sturm A., Essack S. (2006). CTX-M-37 in Salmonella enterica serotype Isangi from Durban, South Africa. Int. J. Antimicrob. Agents.

[4] Suleyman G., Perri M., Vager D., Samuel L., Zervos M.J., Alangaden G., Tibbetts R.J. (2016). Characterization of Salmonella Isangi possessing a CTX-M15 ESBL associated with an outbreak in a US Hospital. Diagn. Microbiol. Infect. Dis..

[5] Li X.-P., Gao R.-H., Hou P.-B., Ren Y.-Y., Zhang H.-N., Jiang K.-Y., Chen Y.-Z., Qi Z.-G., Xu M., Bi Z.-W. (2017). Characterization of the *Salmonella enterica* Serotype Isangi Isolated from Patients for the First Time in China. Foodborne Pathog. Dis..

[6] Kruger T., Szabo D., Keddy K.H., Deeley K., Marsh J.W., Hujer A.M., Bonomo R.A., Paterson D.L. (2004). Infections with Nontyphoidal *Salmonella* Species Producing TEM-63 or a Novel TEM Enzyme, TEM-131, in South Africa. Antimicrob. Agents Chemother..

[7] Hasman H., Mevius D., Veldman K., Olesen I., Aarestrup F.M. (2005). β-Lactamases among extended-spectrum β-lactamase (ESBL)-resistant Salmonella from poultry, poultry products and human patients in The Netherlands. J. Antimicrob. Chemother..

[8] Kulkarni R., Ajantha G., Shubhada C., Jain P. (2009). Isolation of salmonella enterica serotype isangi from a suspected case of enteric encephalopathy. Indian J. Med. Microbiol..

[9] Monte D.F., Nethery M.A., Barrangou R., Landgraf M., Fedorka-Cray P.J. (2021). Whole-genome sequencing analysis and CRISPR genotyping of rare antibiotic-resistant Salmonella enterica serovars isolated from food and related sources. Food Microbiol..

[10] Giardine B., Riemer C., Hardison R.C., Burhans R., Elnitski L., Shah P., Zhang Y., Blankenberg D., Albert I., Taylor J. (2005). Galaxy: A platform for interactive large-scale genome analysis. Genome Res..

[11] Wang B., Ramazzotti D., De Sano L., Zhu J., Pierson E., Batzoglou S. (2018). SIMLR: A Tool for Large-Scale Genomic Analyses by Multi-Kernel Learning. Proteomics.

[12] dos Santos A.M., Panzenhagen P., Ferrari R.G., Conte-Junior C.A. (2022). Large-scale genomic analysis reveals the pESI-like megaplasmid presence in Salmonella Agona, Muenchen, Schwarzengrund, and Senftenberg. Food Microbiol..

[13] dos Santos A.M., Panzenhagen P., Ferrari R.G., Rodrigues G.L., Conte-Junior C.A. (2021). The pESI megaplasmid conferring virulence and multiple-drug resistance is detected in a Salmonella Infantis genome from Brazil. Infect. Genet. Evol..

[14] Rodrigues G.L., Panzenhagen P., Ferrari R.G., dos Santos A., Paschoalin V.M.F., Conte-Junior C.A. (2020). Frequency of Antimi-crobial Resistance Genes in Salmonella From Brazil by in silico Whole-Genome Sequencing Analysis: An Overview of the Last Four Decades. Front. Microbiol..

[15] Panzenhagen P., Portes A.B., dos Santos A.M.P., Duque S.d.S., Junior C.A.C. (2021). The Distribution of *Campylobacter jejuni* Virulence Genes in Genomes Worldwide Derived from the NCBI Pathogen Detection Database. Genes.

[16] Bolger A.M., Lohse M., Usadel B. (2014). Trimmomatic: A flexible trimmer for Illumina sequence data. Bioinformatics.

[17] Bankevich A., Nurk S., Antipov D., Gurevich A.A., Dvorkin M., Kulikov A.S., Lesin V.M., Nikolenko S.I., Pham S., Prjibelski A.D. (2012). SPAdes: A new genome assembly algorithm and its applications to single-cell sequencing. J. Comput. Biol..

[18] Gurevich A., Saveliev V., Vyahhi N., Tesler G. (2013). QUAST: Quality assessment tool for genome assemblies. Bioinformatics.

[19] Rissman A.I., Mau B., Biehl B.S., Darling A.E., Glasner J.D., Perna N.T. (2009). Reordering contigs of draft genomes using the Mauve Aligner. Bioinformatics.

[20] Seemann T. (2014). Prokka: Rapid Prokaryotic Genome Annotation. Bioinformatics.

[21] Yoshida C.E., Kruczkiewicz P., Laing C.R., Lingohr E.J., Gannon V.P.J., Nash J.H.E., Taboada E.N. (2016). The Salmonella In Silico Typing Resource (SISTR): An Open Web-Accessible Tool for Rapidly Typing and Subtyping Draft Salmonella Genome Assemblies. PLoS ONE.

[22] Pearce M.E., Alikhan N.-F., Dallman T.J., Zhou Z., Grant K., Maiden M.C. (2018). Comparative analysis of core genome MLST and SNP typing within a European Salmonella serovar Enteritidis outbreak. Int. J. Food Microbiol..

[23] Zankari E., Allesøe R., Joensen K.G., Cavaco L.M., Lund O., Aarestrup F.M. (2017). PointFinder: A novel web tool for WGS-based detection of antimicrobial resistance associated with chromosomal point mutations in bacterial pathogens. J. Antimicrob. Chemother..

[24] Carattoli A., Zankari E., Garcìa-Fernandez A., Larsen M., Lund O., Voldby Villa L., Møller Aarestrup F., Hasman H. (2014). In Silico Detection and Typing of Plasmids. Antimicrob using PlasmidFinder and plasmid multilocus sequence typing. Agents Chemother..

[25] Page A.J., Taylor B., Delaney A.J., Soares J., Seemann T., Keane J.A., Harris S.R. (2016). SNP-sites: Rapid efficient extraction of SNPs from multi-FASTA alignments. Microb. Genom..

[26] Suleyman G., Tibbetts R., Perri M.B., Vager D., Xin Y., Reyes K., Samuel L., Chami E., Starr P., Pietsch J. (2016). Nosocomial Outbreak of a Novel Extended-Spectrum β-Lactamase *Salmonella enterica* Serotype Isangi Among Surgical Patients. Infect. Control. Hosp. Epidemiol..

[27] Yadava R., Prasad M., Narayan K.G., Jayasheela M., John P.C., Mago M.L., Saxena S.N. (1986). Isolation of Salmonella isangi (6, 7, 14:d:l, 5) for the first time in India. Indian J. Med. Res..

[28] Fassin D., Schneider H. (2003). The politics of AIDS in South Africa: Beyond the controversies. BMJ.

[29] Goldberg M.B., Rubin R.H. (1988). The Spectrum of Salmonella Infection. Infect. Dis. Clin. N. Am..

[30] Bevan E.R., Jones A.M., Hawkey P.M. (2017). Global epidemiology of CTX-M β-lactamases: Temporal and geographical shifts in genotype. J. Antimicrob. Chemother..

[31] Mahérault A.-C., Kemble H., Magnan M., Gachet B., Roche D., Le Nagard H., Tenaillon O., Denamur E., Branger C., Landraud L. (2019). Advantage of the F2:A1:B- IncF Pandemic Plasmid over IncC Plasmids in In Vitro Acquisition and Evolution of *bla*_CTX-M_ Gene-Bearing Plasmids in *Escherichia coli*. Antimicrob. Agents Chemother..

[32] Carattoli A. (2009). Resistance Plasmid Families in *Enterobacteriaceae*. Antimicrob. Agents Chemother..

[33] Leflon-Guibout V., Blanco J., Amaqdouf K., Mora A., Guize L., Nicolas-Chanoine M.-H. (2008). Absence of CTX-M Enzymes but High Prevalence of Clones, Including Clone ST131, among Fecal *Escherichia coli* Isolates from Healthy Subjects Living in the Area of Paris, France. J. Clin. Microbiol..

[34] Poirel L., Bonnin R.A., Nordmann P. (2011). Analysis of the Resistome of a Multidrug-Resistant NDM-1-Producing Escherichia coli Strain by High-Throughput Genome Sequencing. Antimicrob. Agents Chemother..

[35] Briales A., Rodríguez-Martínez J.M., Velasco C., de Alba P.D., Domínguez-Herrera J., Pachón J., Pascual A. (2011). In Vitro Effect of *qnrA1*, *qnrB1*, and *qnrS1* Genes on Fluoroquinolone Activity against Isogenic *Escherichia coli* Isolates with Mutations in *gyrA* and *parC*. Antimicrob. Agents Chemother..

[36] Tran J.H., Jacoby G.A. (2002). Mechanism of plasmid-mediated quinolone resistance. Proc. Natl. Acad. Sci. USA.

[37] Paglietti B., Falchi G., Mason P., Chitsatso O., Nair S., Gwanzura L., Uzzau S., Cappuccinelli P., Wain J., Rubino S. (2013). Diversity among human non-typhoidal salmonellae isolates from Zimbabwe. Trans. R. Soc. Trop. Med. Hyg..

[38] Yue L., Jiang H.-X., Liao X.-P., Liu J.-H., Li S.-J., Chen X.-Y., Chen C.-X., Lü D.-H., Liu Y.-H. (2008). Prevalence of plasmid-mediated quinolone resistance qnr genes in poultry and swine clinical isolates of Escherichia coli. Veter. Microbiol..

[39] Singer A.C., Shaw H., Rhodes V., Hart A. (2016). Review of Antimicrobial Resistance in the Environment and Its Relevance to Environmental Regulators. Front. Microbiol..

[40] Ferrari R., Galiana A., Cremades R., Rodriguez J.C., Magnani M., Tognim M.C.B., Oliveira T.C.R.M., Royo G. (2011). Plasmid-mediated quinolone resistance by genes qnrA1 and qnrB19 in Salmonella strains isolated in Brazil. J. Infect. Dev. Ctries.

[41] García-Fernández A., Fortini D., Veldman K., Mevius D., Carattoli A. (2009). Characterization of plasmids harbouring qnrS1, qnrB2 and qnrB19 genes in Salmonella. J. Antimicrob. Chemother..

[42] Tyson G.H., Li C., Hsu C.-H., Bodeis-Jones S., McDermott P.F. (2019). Diverse Fluoroquinolone Resistance Plasmids From Retail Meat E. coli in the United States. Front. Microbiol..

[43] Wasyl D., Hoszowski A., Zając M. (2014). Prevalence and characterisation of quinolone resistance mechanisms in *Salmonella* spp.. Veter. Microbiol..

[44] González F., Araque M. (2013). Association of Transferable Quinolone Resistance Determinant *qnrB19* with Extended-Spectrum*β*-Lactamases in *Salmonella* Give and *Salmonella* Heidelberg in Venezuela. Int. J. Microbiol..

[45] Karczmarczyk M., Martins M., McCusker M., Mattar S., Amaral L., Leonard N., Aarestrup F.M., Fanning S. (2010). Characterization of antimicrobial resistance in Salmonella enterica food and animal isolates from Colombia: Identification of a qnrB19-mediated quinolone resistance marker in two novel serovars. FEMS Microbiol. Lett..

[46] Jibril A.H., Okeke I.N., Dalsgaard A., Menéndez V.G., Olsen J.E. (2021). Genomic Analysis of Antimicrobial Resistance and Resistance Plasmids in *Salmonella* Serovars from Poultry in Nigeria. Antibiotics.

[47] Veldman K., Cavaco L.M., Mevius D., Battisti A., Franco A., Botteldoorn N., Bruneau M., Perrin-Guyomard A., Cerny T., Escobar C.D.F. (2011). International collaborative study on the occurrence of plasmid-mediated quinolone resistance in Salmonella enterica and Escherichia coli isolated from animals, humans, food and the environment in 13 European countries. J. Antimicrob. Chemother..

[48] Fitch F.M., Carmo-Rodrigues M.S., Oliveira V.G.S., Gaspari M.V., dos Santos A., de Freitas J.B., Pignatari A.C. (2016). β-Lactam Resistance Genes: Characterization, Epidemiology, and First Detection of *bla*_CTX-M-1_ and *bla*_CTX-M-14_ in *Salmonella* spp. Isolated from Poultry in Brazil—Brazil Ministry of Agriculture’s Pathogen Reduction Program. Microb. Drug Resist..

[49] Fernandes S.A., Camargo C.H., Francisco G.R., Bueno M.F.C., Garcia D.O., Doi Y., Casas M.R.T. (2017). Prevalence of Extended-Spectrum β-Lactamases CTX-M-8 and CTX-M-2-Producing *Salmonella* Serotypes from Clinical and Nonhuman Isolates in Brazil. Microb. Drug Resist..

[50] Castellanos L.R., Van Der Graaf-Van Bloois L., Donado-Godoy P., León M., Clavijo V., Arévalo A., Bernal J.F., Mevius D.J., Wagenaar J.A., Zomer A. (2018). Genomic Characterization of Extended-Spectrum Cephalosporin-Resistant Salmonella enterica in the Colombian Poultry Chain. Front. Microbiol..

[51] Perin A.P., Martins B.T.F., Barreiros M.A.B., Yamatogi R.S., Nero L.A., Bersot L.d.S. (2019). Occurrence, quantification, pulse types, and antimicrobial susceptibility of Salmonella sp. isolated from chicken meat in the state of Paraná, Brazil. Braz. J. Microbiol..

[52] Bartoloni A., Pallecchi L., Riccobono E., Mantella A., Magnelli D., Di Maggio T., Villagran A., Lara Y., Saavedra C., Strohmeyer M. (2013). Relentless increase of resistance to fluoroquinolones and expanded-spectrum cephalosporins in Escherichia coli: 20 years of surveillance in resource-limited settings from Latin America. Clin. Microbiol. Infect..

[53] Leão C., Clemente L., Moura L., Seyfarth A.M., Hansen I.M., Hendriksen R.S., Amaro A. (2021). Emergence and Clonal Spread of CTX-M-65-Producing Escherichia coli From Retail Meat in Portugal. Front. Microbiol..

[54] Furlan J.P.R., Lopes R., Ramos M.S., dos Santos L.D.R., Rosa R.d.S., Savazzi E.A., Stehling E.G. (2021). Colistin-resistant mcr-1-positive Escherichia coli ST1775-H137 co-harboring blaCTX-M-2 and blaCMY-2 recovered from an urban stream. Infect. Genet. Evol..

[55] Palmeira J.D., Ferreira H., Madec J.-Y., Haenni M. (2018). Draft genome of a ST443 mcr-1—And bla CTX-M-2 -carrying Escherichia coli from cattle in Brazil. J. Glob. Antimicrob. Resist..

[56] Hall J.P.J., Botelho J., Cazares A., Baltrus D.A. (2021). What makes a megaplasmid?. Philos. Trans. R. Soc. B Biol. Sci..

[57] Petermann S.R., Sherwood J.S., Logue C.M. (2008). The Yersinia high pathogenicity island is present in Salmonella enterica Subspecies I isolated from turkeys. Microb. Pathog..

[58] dos Santos A.M., Ferrari R.G., Panzenhagen P., Rodrigues G.L., Conte-Junior C.A. (2021). Virulence genes identification and characterization revealed the presence of the Yersinia High Pathogenicity Island (HPI) in Salmonella from Brazil. Gene.

[59] Oelschlaeger T.A., Zhang D., Schubert S., Carniel E., Rabsch W., Karch H., Hacker J. (2003). The High-Pathogenicity Island Is Absent in Human Pathogens of *Salmonella enterica* Subspecies I but Present in Isolates of Subspecies III and VI. J. Bacteriol..

